# The Health Effects of Passive Smoking: An Overview of Systematic Reviews Based on Observational Epidemiological Evidence

**DOI:** 10.1371/journal.pone.0139907

**Published:** 2015-10-06

**Authors:** Shiyi Cao, Chen Yang, Yong Gan, Zuxun Lu

**Affiliations:** School of Public Health, Tongji Medical College, Huazhong University of Science and Technology, Wuhan, China; Shanghai Institute of Hypertension, CHINA

## Abstract

**Purpose:**

We aim to systematically summarize the available epidemiological evidence to identify the impact of environmental tobacco smoke on health.

**Methods:**

A systematic literature search of PubMed, Embase, Web of Science, and Scopus for meta-analyses was conducted through January 2015. We included systematic reviews that investigated the association between passive smoking and certain diseases. Quantitative outcomes of association between passive smoking and the risk of certain diseases were summarized.

**Results:**

Sixteen meta-analyses covering 130 cohort studies, 159 case-control studies, and 161 cross-sectional studies and involving 25 diseases or health problems were reviewed. Passive smoking appears not to be significantly associated with eight diseases or health problems, but significantly elevates the risk for eleven specific diseases or health problems, including invasive meningococcal disease in children (OR 2.18; 95% CI 1.63–2.92), cervical cancer (OR 1.73; 95% CI 1.35–2.21), Neisseria meningitidis carriage (OR 1.68; 95% CI 1.19–2.36), Streptococcus pneumoniae carriage (OR 1.66; 95% CI 1.33–2.07), lower respiratory infections in infancy (OR 1.42; 95% CI 1.33–1.51), food allergy (OR 1.43; 95% CI 1.12–1.83), and so on.

**Conclusions:**

Our overview of systematic reviews of observational epidemiological evidence suggests that passive smoking is significantly associated with an increasing risk of many diseases or health problems, especially diseases in children and cancers.

## Introduction

Smoking is a major public health problem worldwide. There have been thousands of studies investigating the impact of active smoking on health, and the overall toxic effects of active smoking are generally recognized [1]. In comparison, the effects of passive smoking on health are not fully understood. Existing studies suggest that passive smoking and active smoking might equally increase the risk of certain diseases, such as female breast cancer [2], allergic rhinitis, allergic dermatitis, and food allergy [3]. As early as 1928, Schonherr suspected that inhalation of husbands’ smoke could cause lung cancer among non-smoking wives [4]. Since then a substantial body of research about environmental tobacco smoke and health has appeared [5]. But the impact of passive smoking on health remains largely inconclusive and has not been systematically summarized.

Due to the relative small health risks associated with exposure to passive smoking, investigation of this issue requires large study sizes. Difficulties in measuring passive smoking and controlling various confounding factors further add to the uncertainty in any investigation of the effects of passive smoking. Consequently, a meta-analysis, pooling together individual original studies quantitatively, has played an important part in establishing the evidence about the health effects of passive smoking [5]. Since Zmirou evaluated the respiratory risk of passive smoking by a meta-analysis in the early 1990s, many meta-analyses of observational epidemiological studies have been published to identify the impact of passive smoking on health.

Recognizing that the evidence is accumulating constantly worldwide, we conducted an overview of systematic reviews that have summarized the evidence from observational epidemiological studies on the health effects of passive smoking.

## Methods

No protocol exists for this overview of systematic reviews.

### Ethics

Data for this research was acquired from previously published papers. Written consent and ethical approval were not required.

### Literature search strategy

We attempted to conduct this overview of systematic reviews in accordance with the rationale and guideline recommended by Cochrane handbook 5.1.0 [6] (S1 Checklist). A systematic literature search of PubMed, Embase, Web of Science, and Scopus was conducted in January 2015 using the following search terms with no restrictions: passive smoking, secondhand smoking, environmental tobacco smoke, involuntary smoking, and tobacco smoke pollution. The reference lists of the retrieved articles were also reviewed. We did not contact authors of the primary studies for additional information.

### Selection of relevant systematic reviews

Systematic reviews meeting the following criteria were regarded as eligible: (1) the design was meta-analysis, (2) passive smoking was an exposure variable and the outcome was the incidence of certain diseases or health problems, (3) the included original studies were cross-sectional, case-control, or/and cohort study design, (4) the literature search was international or worldwide, and (5) the pooled relative risk (RR) or odds ratio (OR) and the corresponding 95% confidence interval (CI) of specific diseases relating to exposure to passive smoking were reported or could be calculated from the data provided. Systematic reviews in which all included original studies were conducted in one country or region were excluded. We also excluded the meta-analyses that investigated the association between maternal smoking in pregnancy and the health risk of offspring. All potential meta-analyses were independently screened by two authors (SC and CY), who reviewed the titles or/abstracts first and then conducted a full-text assessment. Disagreements between the two reviewers were resolved through discussion with the third investigator (ZL).

### Data extraction

The following information was extracted from the studies by two investigators (SC and CY): first author, publication year, country, number and design of the included original studies, and main quantitative estimates of the association of interest.

### Quality appraisal

We appraised all the included meta-analyses using the Assessment of Multiple Systematic Reviews (AMSTAR) standard, an 11-item assessment tool designed to appraise the methodological quality of systematic reviews [7]. The maximum score is 11, and 0–4, 5–8, and 9–11 respectively indicates low, moderate, and high quality [8]. Disagreements on assessment scores were resolved by discussion among the authors.

### Synthesis of the evidence

There may be more than one meta-analysis published regarding the association between passive smoking and risk of a specific disease. We only included the latest meta-analysis and excluded all the previous ones. For each included meta-analysis, we summarized the number and design of the included original studies, the main quantitative estimates of association of interest, heterogeneity between original studies, and so on. In any included meta-analyses, when estimates of association between passive smoking and certain diseases were reported separately for subgroups, we combined the results of the subgroups and calculated common estimates using a fixed-effects model if appropriate.

## Results

### Literature search

**Fig 1:**
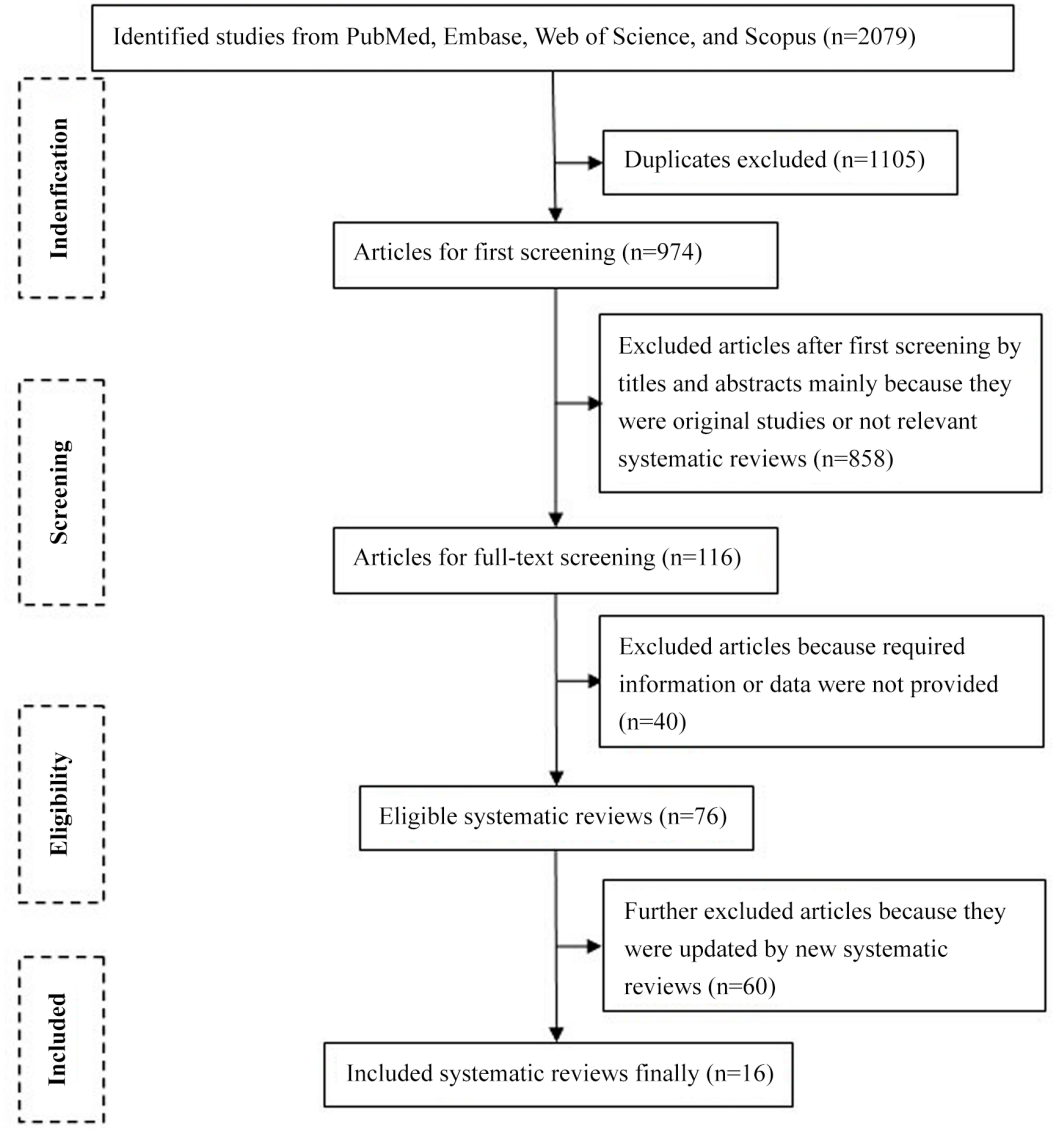
Identification of relevant meta-analyses.

Fig 1 shows the process of study identification and inclusion. Initially, we retrieved 2,079 articles from Pubmed, Emabse, Web of Science, and Scopus. After 1,105 duplicates were excluded, 974 articles were screened through titles and abstracts, of which 858 were excluded mainly because they were original studies or irrelevant reviews. After full-text review of the remaining 116 articles, 100 were further excluded because they did not report the outcomes of interest or their findings were already updated by newer systematic reviews. Finally, 16 meta-analyses were included [3, 9–23].

### Characteristics and quality of the included systematic reviews

The main characteristics of the sixteen meta-analyses were summarized in Table 1. These meta-analyses covered a total of 130 cohort studies, 159 case-control studies, and 161 cross-sectional studies. They were published between 1998 and 2014. The quality scores of these meta-analyses appraised using AMSTAR ranged from 3 to 10. The numbers of meta-analyses with high quality, middle quality, and low quality were 5, 9, and 2 respectively (see Table 2).

**Table 1:** Main characteristics of the included systematic reviews.

Author	Year	Diseases	Number and design of included studies	Pooled odds ratio (95% confidence interval)
Lee CC	2010	pediatric invasive bacterial disease and bacterial carriage	30 case-control studies for invasive bacterial disease	invasive bacterial disease:
			12 cross-sectional studies for bacterial carriage	1.21 (95% CI 0.69–2.14) for invasive pneumococcal disease
				1.22 (95% CI 0.93–1.62) for invasive Hib disease.
				pharyngeal carriage:
				1.68 (95% CI, 1.19–2.36) for Neisseria. meningitidies
				1.66 (95% CI 1.33–2.07) for Streptococcus. pneumoniae
				0.96 (95% CI 0.48–1.95) for Hib.
Van Hemelrijck MJ,	2009	bladder cancer	8 studies	0.99 (95% CI 0.86–1.14)
			3 cohort	1.19 (95% CI 0.88–1.62) for childhood passive smoking
			5 case-control	0.90 (95% CI 0.79–1.02) for adulthood passive smoking
Jones DT	2008	inflammatory bowel disease	13 case-control studies	1.10 (95% CI 0.92–1.30) for Crohn's disease
				1.01 (95% CI 0.85–1.20) for ulcerative colitis
Jones LL	2011	lower respiratory infections in infancy	60 studies	1.22 (95% CI 1.10–1.35) for paternal smoking
			32 cohort	1.62 (95% CI 1.38–1.89) for both parents smoking
			15 case-control	1.54 (95% CI 1.40–1.69) for any household member smoking.
			13 cross-sectional	
Zeng XT	2012	cervical cancer	11 case-control studies	1.73 (95% CI 1.35–2.21)
Strachan DP	1998	middle ear disease in children	28 studies	1.48 (95% CI 1.08–2.04) for recurrent otitis media,
			11 cohort	1.38 (95% CI 1.23–1.55) for middle ear effusion
			13 case-control	1.21 (95% CI 0.95–1.53) for glue ear.
			4 cross-sectional	
Murray RL	2012	invasive meningococcal disease in children	18 studies	2.18 (95% CI 1.63–2.92)
			2 cohort	2.48 (95% CI 1.51–4.09) in children under 5 years.
			16 case-control	2.26 (95% CI 1.54–3.31) for maternal smoking after birth
Zhou J	2012	pancreatic cancer	10 studies	1.12 (95% CI 0.89–1.43) during childhood.
			7 cohort	1.23 (95% CI 0.86–1.77) during adulthood at home
			3 case-control	0.94 (95% CI 0.67–1.33) during adulthood at work
Lin HH	2007	tuberculosis	4 case-control studies	4.01 (95% CI 2.54–6.34)
Saulyte J	2014	allergic rhinitis, allergic dermatitis, and food allergy in adults and children	63 studies for allergic rhinitis	1.10 (95% CI 1.06–1.15)
			9 cohort	1.07 (95% CI 1.03–1.12) for allergic dermatitis
			3 case-control	1.09 (95% CI 1.04–1.14) for allergic rhinitis
			51 cross-sectional	1.43 (95% CI 1.12–1.83) for food allergy
			58 studies for allergic dermatitis	
			14 cohort	
			5 case-control	
			39 cross-sectional	
			6 studies for food allergies	
			5 cohort	
			1 cross-sectional	
Sun K	2014	diabetes	6 cohort studies	1.21 (95% CI 1.07–1.38)
Oono IP	2011	stroke	20 studies	1.25 (95% CI 1.12–1.38)
			10 cohort	1.22 (95% CI 1.08–1.38) for cohort studies
			6 case-control	1.41 (95% CI 1.15–1.72) for case-control studies
			4 cross-sectional	1.03 (95% CI 0.69–1.53) for cross-sectional studies
Tinuoye O	2013	physician-diagnosed childhood asthma	20 studies	1.32 (95% CI 1.23–1.42)
			4 cohort	1.26 (95% CI 0.91–1.73) for cohort studies
			2 case-control	1.41 (95% CI 1.31–2.32) for case-control studies
			14 cross-sectional	1.31 (95% CI 1.22–1.43) for cross-sectional studies
Yang Y	2013	breast cancer	10 cohort studies.	1.01 (95% CI 0.96–1.06)
				0.96 (95% CI 0.81–1.14) for passive smoking at home
				1.01 (95% CI 0.93–1.10) for passive smoking in the workplace
He J	1999	coronary heart disease	18 studies	1.25 (95% CI 1.17–1.32)
			10 cohort	1.17 (95% CI 1.11–1.24) for passive smoking at home
			8 case-control	1.11 (95% CI 1.00–1.23) for passive smoking in the workplace
Taylor R	2007	lung cancer	55 studies	1.27 (95% CI 1.17–1.37)
			7 cohort	1.15 (95% CI 1.03–1.28) for North America
			25 population-based case-control	1.31 (95% CI 1.16–1.48) for Asia
			23 non-population-based case-control	1.31 (95% CI 1.24–1.52) for Europe

**Table 2:** Appraisal of the included meta-analyses on the impact of passive smoking on various diseases.

Author	‘A priori’ design provided	Duplicate study selection/data extraction	Comprehensive literature search	Status of publication as inclusion criteria	List of studies included/excluded provided	Characteristics of included studies documented	Scientific quality assessed and documented	Appropriate formulation of conclusions	Appropriate methods of combining studies;	Assessment of publication bias; and	Conflict of interest statement.	Total yes
Lee CC	yes	yes	yes	no	yes	yes	no	yes	yes	yes	yes	9
Van H MJ,	no	no	no	yes	yes	yes	no	no	yes	yes	yes	6
Jones DT	yes	yes	yes	no	yes	yes	yes	no	yes	yes	yes	9
Jones LL	yes	yes	yes	no	yes	no	yes	yes	yes	yes	yes	9
Zeng XT	no	yes	no	yes	yes	yes	yes	no	yes	yes	no	7
Strachan DP	no	no	yes	no	no	yes	no	no	yes	no	no	3
Murray RL	yes	yes	yes	no	yes	yes	no	no	yes	yes	yes	8
Zhou J	no	no	yes	no	no	yes	no	no	yes	yes	yes	5
Lin HH	no	yes	no	no	yes	yes	yes	yes	yes	yes	yes	8
Saulyte J	no	yes	yes	no	yes	yes	yes	no	yes	yes	yes	8
Sun K	no	no	yes	no	yes	yes	yes	no	yes	no	yes	6
Oono IP	no	yes	no	no	yes	yes	no	yes	yes	yes	no	6
Tinuoye O	no	no	yes	no	no	yes	no	no	yes	yes	yes	5
Yang Y	yes	yes	yes	no	yes	yes	yes	yes	yes	yes	yes	10
He J	no	yes	yes	no	no	yes	no	no	no	yes	no	4
Taylor R	yes	yes	yes	yes	yes	yes	no	yes	yes	yes	yes	10

### The Main Health Consequences of Passive Smoking

**Fig 2:**
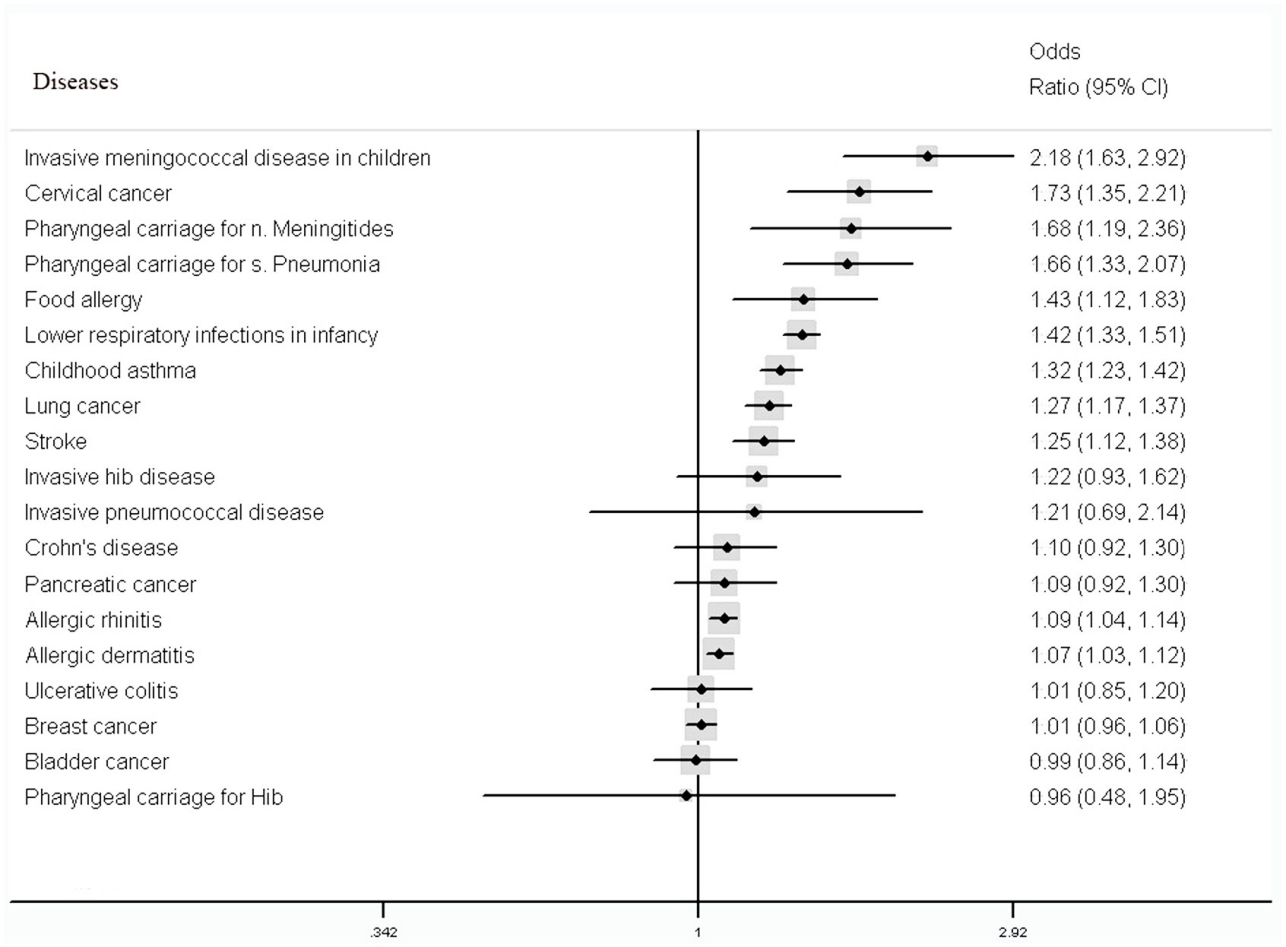
Summary of estimates of associations between passive smoking and the risk of specific diseases or health problems.

Fig 2 shows the integrated results on the impact of passive smoking on specific diseases. The included 16 meta-analyses covered 25 diseases or health problems. There was statistically significant positive relationship between exposure environmental tobacco smoke and the risk of eleven diseases, especially invasive meningococcal disease in children (OR 2.18; 95% CI 1.63–2.92) and other three diseases or health problems with a 1.5 to 2.0-fold increase in the risk: cervical cancer (OR 1.73; 95% CI 1.35–2.21), Neisseria meningitidis carriage (OR 1.68; 95% CI 1.19–2.36), and Streptococcus pneumoniae carriage (OR 1.66; 95% CI 1.33–2.07). The increase in the risk of other seven diseases associated with exposure to passive smoking was statistically significant but small in impact size (OR was less than 1.5): lower respiratory infections in infancy (OR 1.42; 95% CI 1.33–1.51), food allergy (OR 1.43; 95% CI 1.12–1.83), childhood asthma (OR 1.32; 95% CI 1.23–1.42), lung cancer (OR 1.27; 95% CI 1.17–1.37), stroke (OR 1.25; 95% CI 1.12–1.38), allergic rhinitis (OR 1.09; 95% CI 1.04–1.14), and allergic dermatitis (OR 1.07; 95% CI 1.03–1.12). Of these 25 diseases or health problems, eight diseases were not found to be significantly associated with passive smoking. They were invasive Haemophilus influenzae type B (Hib) disease, invasive pneumococcal disease, Crohn's disease, pancreatic cancer, ulcerative colitis, breast cancer, bladder cancer, and pharyngeal carriage for Hib. In addition, the effects of passive smoking on increased risk of coronary heart disease, tuberculosis, diabetes, and middle ear disease in children (recurrent otitis media, middle ear effusion, and glue ear) were not conclusive, because the number of included studies was small or the quality of the corresponding meta-analysis was low.

### Passive smoking and cancer risk

We investigated the association of passive smoking with the risk of lung cancer, cervical cancer, pancreatic cancer, breast cancer, and bladder cancer. Based on 55 observational studies (7 cohort studies, 25 population-based case-control studies and 23 non-population-based case-control studies), passive smoking were found to be associated with the increased risk of lung cancer (OR 1.27; 95% CI 1.17 to 1.37). The ORs for lung cancer in North America, Asia, and Europe were similar [19]. 11 case-control studies, involving 3,230 cases and 2,982 controls, suggested a positive relationship between passive smoking and cervical cancer (OR 1.73; 95% CI 1.35–2.21) [15]. Pancreatic cancer [21], breast cancer [13], and bladder cancer were not found to be associated with passive smoking.

### Passive smoking and allergic diseases

A meta-analysis of observational studies published in PLOS Medicine systematically reviewed the effects of exposure to environmental smoke on allergic diseases [3]. The pooled ORs of 63 studies for allergic rhinitis, 58 studies for allergic dermatitis, and 6 studies for food allergies were 1.07 (95% CI 1.03–1.12), 1.09 (95% CI 1.04–1.14), and 1.43 (95% CI 1.12–1.83) respectively. Another meta-analysis investigated the association between passive smoking and the risk of physician-diagnosed childhood asthma [9], and suggested that there was consistent evidence of a modest positive association between them (OR 1.32; 95% CI: 1.23–1.42).

### Passive smoking and pediatric invasive bacterial disease and bacterial carriage

Passive smoking was also thought to be associated with pediatric invasive bacterial disease and bacterial carriage. A meta-analysis involving 30 case-control studies for invasive bacterial disease and 12 cross-sectional studies for bacterial carriage indicated that the risk of invasive meningococcal disease, pharyngeal carriage for Neisseria, meningitidies and Streptococcus pneumoniae were significantly associated with passive smoking, and the ORs were 2.18, 95% CI 1.63 to 2.92), 1.68 (95% CI, 1.19–2.36), and 1.66 (95% CI 1.33–2.07), respectively. The risk of invasive pneumococcal disease, invasive Hib disease, and pharyngeal carriage for Hib were not found to be related to exposure to environmental smoke.

## Discussion

The health effects of environmental tobacco smoke are attracting more and more attention worldwide. Increasing numbers of original studies and meta-analyses are being published focusing on this important issue. In the present overview of systematic reviews based on sixteen systematic reviews involving 450 original observational studies, we found that passive smoking could significantly increase the risk of eleven diseases, especially invasive meningococcal disease in children, cervical cancer, Neisseria meningitidis carriage, and Streptococcus. pneumoniae carriage, but not associated with other eight diseases. Cancers were one of the most common investigated health outcomes associated with passive smoking. We found that exposure to environmental tobacco smoke could increase the risk of lung cancer and cervical cancer, but was not the risk of pancreatic cancer, breast cancer, or bladder cancer. It appears that passive smoking could increase the risk of some diseases among children, especially bacterial infections (e.g., lower respiratory infections in infancy, middle ear disease in children, invasive meningococcal disease in children, allergic diseases in children, and childhood asthma).

Previously, there were some reviews focusing on the health effects of exposure to environmental tobacco smoke. But they were qualitative or only involved children or limited to several diseases [24–26]. We used a systematic overview to summarize the quantitative estimates of the associations between passive smoking and various diseases based on all latest available meta-analyses. It should be noted that, in the present overview, we excluded meta-analyses evaluating the effects of smoking during pregnancy on fetus or offspring health, because the effects was obviously different from the health effects of active smoking or conventional passive smoking in the general population.

The quality of included original studies influences the reliability of the results and conclusions of the corresponding meta-analysis; similarly, the validity of the results of an overview of systematic reviews depends on the quality of the included systematic reviews. We used AMSTAR protocol, an internationally recognized assessment tool, to appraise the methodological quality of all included meta-analyses, and found that there were two meta-analyses with low quality. Accordingly, the conclusions drawn based on these two meta-analyses involving middle ear disease in children and coronary heart disease need to be interpreted with caution.

The evidence level of meta-analyses partly depends on the number and the design type of included original studies. Although there was no consensus about the minimum number of original studies included in meta-analysis, but more caution is needed when an association is assessed based on a small number of original studies. In our overview, we found a significant positive association between passive smoking and tuberculosis (OR 4.01; 95% CI 2.54–6.34), but it was only based on 4 case-control studies. More studies should be conducted to further assess the relationship between them. Similarly, the effect of passive smoking on diabetes was based on 6 cohort studies (OR 1.21; 95% CI 1.07–1.38), and more original studies are also needed.

There were several strengths in our research. Firstly, we followed the primary rationale and method of Cochrane overviews of reviews [6] to summarize the health consequences of certain exposure. Overview of systematic reviews is primarily intended to summarize multiple reviews addressing the effects of two or more potential interventions for a single condition or health problem. Up to now, most of overviews have been conducted to evaluate the effects of several interventions [27, 28], and very few overviews have addressed the effects of a single exposure factor on multiple diseases or health problems based on observational studies. Our present overview expands the application of overviews of systematic reviews. Additionally, our study provides robust and comprehensive scientific information for smoking ban in public places and for educational pamphlets about passive smoking.

Some limitations in our overview should be noted. Firstly, we only included systematic reviews but not original studies. The associations of passive smoking with some diseases might have been investigated by original studies but not synthesized by meta-analyses and, therefore, were not summarized in this overview. Secondly, the mechanism on the health effects of passive smoking was not be examined since our study only intended to summarize relevant observational epidemiological evidence.

In summary, our overview of systematic reviews of up-to-date epidemiological evidence suggests that passive smoking is significantly associated with an increasing risk of many diseases and health problems, especially diseases in children and cancers. This study provides comprehensive population-based evidence about toxic effect of exposure to environmental tobacco smoke and should benefit developing health promotion strategies of smoking control. Stricter regulations against cigarette smoking should be formulated and implemented, because smoking harms not only own health but also the health of neighboring people.

## Supporting Information

S1 PRISMA Checklist (DOC)
